# How does a nature-based solution for flood control compare to a technical solution? Case study evidence from Belgium

**DOI:** 10.1007/s13280-021-01548-4

**Published:** 2021-05-11

**Authors:** Francis Turkelboom, Rolinde Demeyer, Liesbet Vranken, Piet De Becker, Filip Raymaekers, Lieven De Smet

**Affiliations:** 1 Research team Nature & Society, Institute of Nature and Forest Research (INBO), Havenlaan 88 bus 73, 1000 Brussel, Belgium; 2 Op de Groei, Rijweg 124, 3020 Herent, Belgium; 3 Agricultural and Resource Economics, Department of Earth and Environmental Sciences, KU Leuven, Celestijnenlaan 200 E - Box 2411, 3001 Leuven Heverlee, Belgium; 4 Research team Environment & Climate, Institute of Nature and Forest Research (INBO)., Havenlaan 88 bus 73, 1000 Brussel, Belgium; 5 Section Demer, Dijle and Maas, Flanders Environment Agency (VMM), VAC Dirk Bouts, Diestsepoort 6 bus 73, 3000 Leuven, Belgium

**Keywords:** Biodiversity, Ecosystem services, Flood control, Nature-based solution, Social cost–benefit analysis, Storm basins

## Abstract

The strategy of reconnecting rivers with their floodplains currently gains popularity because it not only harnesses natural capacities of floodplains but also increases social co-benefits and biodiversity. In this paper, we present an example of a successfully implemented nature-based solution (NBS) in the Dijle valley in the centre of Belgium. The research objective is to retrospectively assess cost and benefit differences between a technical solution (storm basins) and an alternative NBS, here the restoration of the alluvial floodplain. The method is a comparative social cost–benefit analysis. The case study analysis reveals similar flood security, lower costs, more ecosystem services benefits and higher biodiversity values associated with the NBS option in comparison to the technical alternative. However, the business case for working with NBS depends substantially on the spatial and socio-ecological context. Chances for successful NBS implementation increase in conditions of sufficient space to retain flood water, when flood water is of sufficient quality, and when economic activity and housing in the floodplain is limited.

**Supplementary Information:**

The online version of this article contains supplementary material available at (10.1007/s13280-021-01548-4).

## Introduction

For centuries, floodplains have been modified and rivers regulated by the construction of channels and dams to enable agricultural production, to protect settlements against flooding, to enhance navigation or to produce energy (Buijse et al. 2002; Posthumus et al. 2010). In Europe and North America, up to 90% of floodplains are already ‘cultivated’ and, therefore, functionally extinct (Tockner and Stanford 2002). In Germany, a survey of the 79 largest rivers showed that only around 35% of the morphological floodplains still serve for natural flood retention. A further decline between 2010 and 2015 was mainly caused by an increase in settlements and transport infrastructure (Walz et al. 2019). While simplification of formerly complex, irregular banks and beds, into straight, uniform (shipping) channels have led to generally more uniform flow conditions, constant water tables and sharply defined embankments, they have given rise to several unintended challenges for society, for instance exacerbating flood risks, diminishing water quality, decreased ecological functioning, biodiversity loss and loss of cultural services related to rivers (such as mental connection to rivers, fishing, water supply, swimming and other recreation activities) (Malmqvist and Rundle 2002; Liao 2014; Kondolf and Pinto 2016; Wantzen 2016).

Well-designed, nature-based solutions (NBS, Nesshöver et al. 2017; EC 2020) are suggested as sustainable ways for addressing water-related risks, as they need less maintenance, are more cost effective, create co-benefits for people, and support high levels of biological diversity today and in the future (Opperman et al. 2009; Halbac-Cotoara-Zamfir 2019; Albert et al. 2019). Floodplain restoration is an example of NBS that can make a significant contribution to a more effective flood risk management, to strengthen multifunctionality of the river landscape and to increase the supply of ecosystem services, although floodplain restoration might not completely eliminate floodings in an era of climate change (Schindler et al. 2014; Kiedrzynska et al. 2015). However, substantial knowledge gaps regarding NBS in river landscapes still exist, particularly related to planning and implementation practices, effectiveness and monitoring, as well as on governance aspects (Albert et al., 2019). Therefore, these authors propose a research and experimentation agenda focussing on the following: (1) effectiveness of NBS, including assessments of the outcomes of both NBS and technical alternatives, (2) co-benefits and costs of NBS using multimetric indicators such as recreation potential, water retention and biodiversity and (3) useful approaches for informed co-design of NBS. In this paper, we present a successful implementation of a NBS in a river landscape in Belgium, where the river was reconnected to its alluvial floodplain in order to ensure flood protection to a nearby city. The objectives of this paper are to assess retrospectively the differences in the costs and benefits between a technical solution for flood control and an alternative NBS, and to describe the process that preceded the decision-making. Based on this experience, implications for policy and floodplain management are formulated.

## Case study description

### The study area south of Leuven

**Fig. 1:**
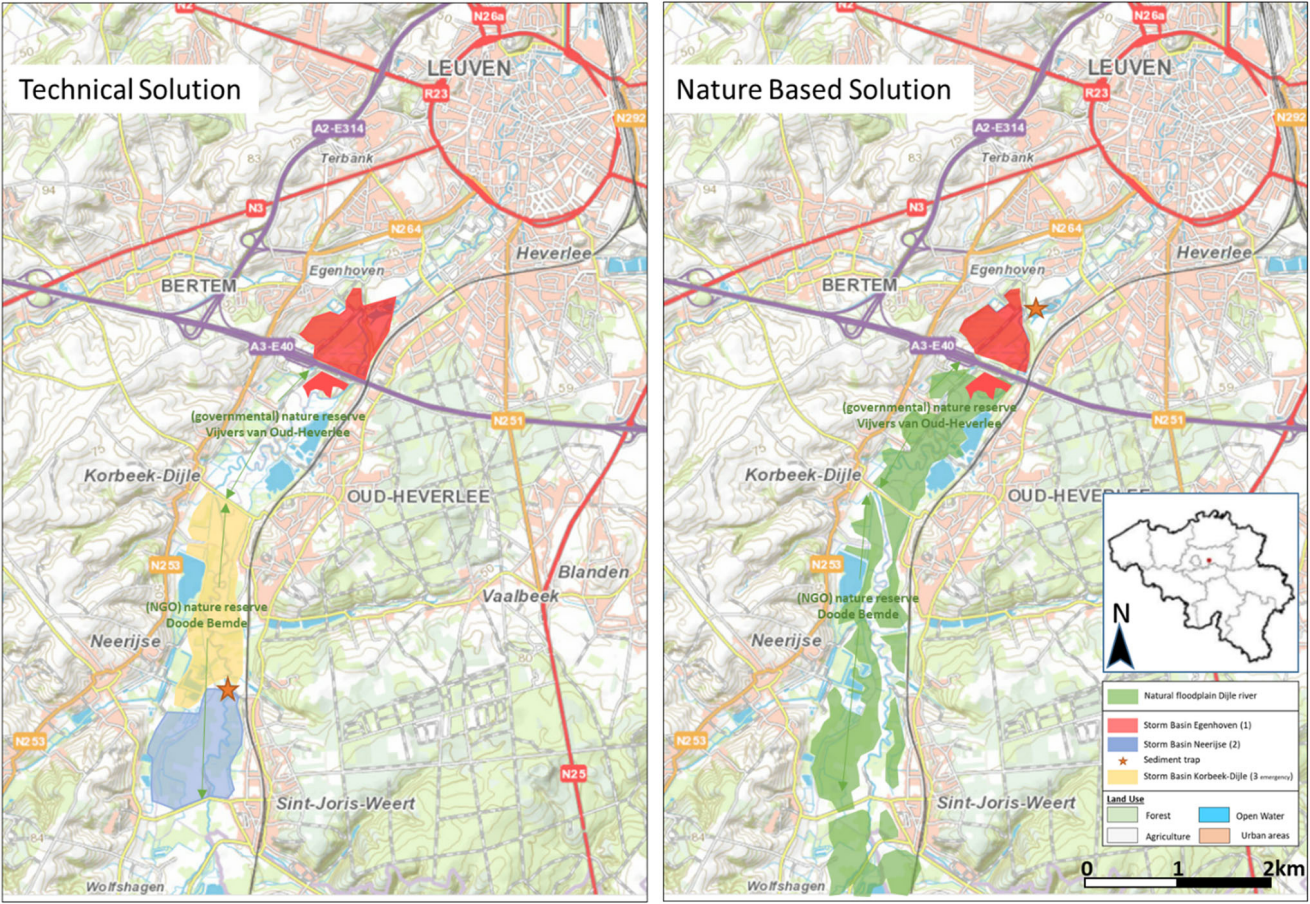
Situation map of the study area with technical solution (left) and nature-based solution (right) for flood risk prevention of the city of Leuven (Belgroma 1990; flooded floodplain area mapped in situ by INBO during 1990-1994)

The catchment of the Dijle river is situated in the central part of Belgium, draining part of a fertile silt plateau (about 100 m above sea level). The river has dug itself some 60–70 m deep in this plateau forming a marked 1-kilometre-wide valley. The study area is situated upstream (south) of the city of Leuven and has a surface of about 800 ha (Fig. 1). The Dijle river is strongly meandering (sinuosity ~1.4–1.8) where the meanders spontaneously move each year 1–1.2 m downstream (Vandaele et al. 2002).

**Fig. 2:**
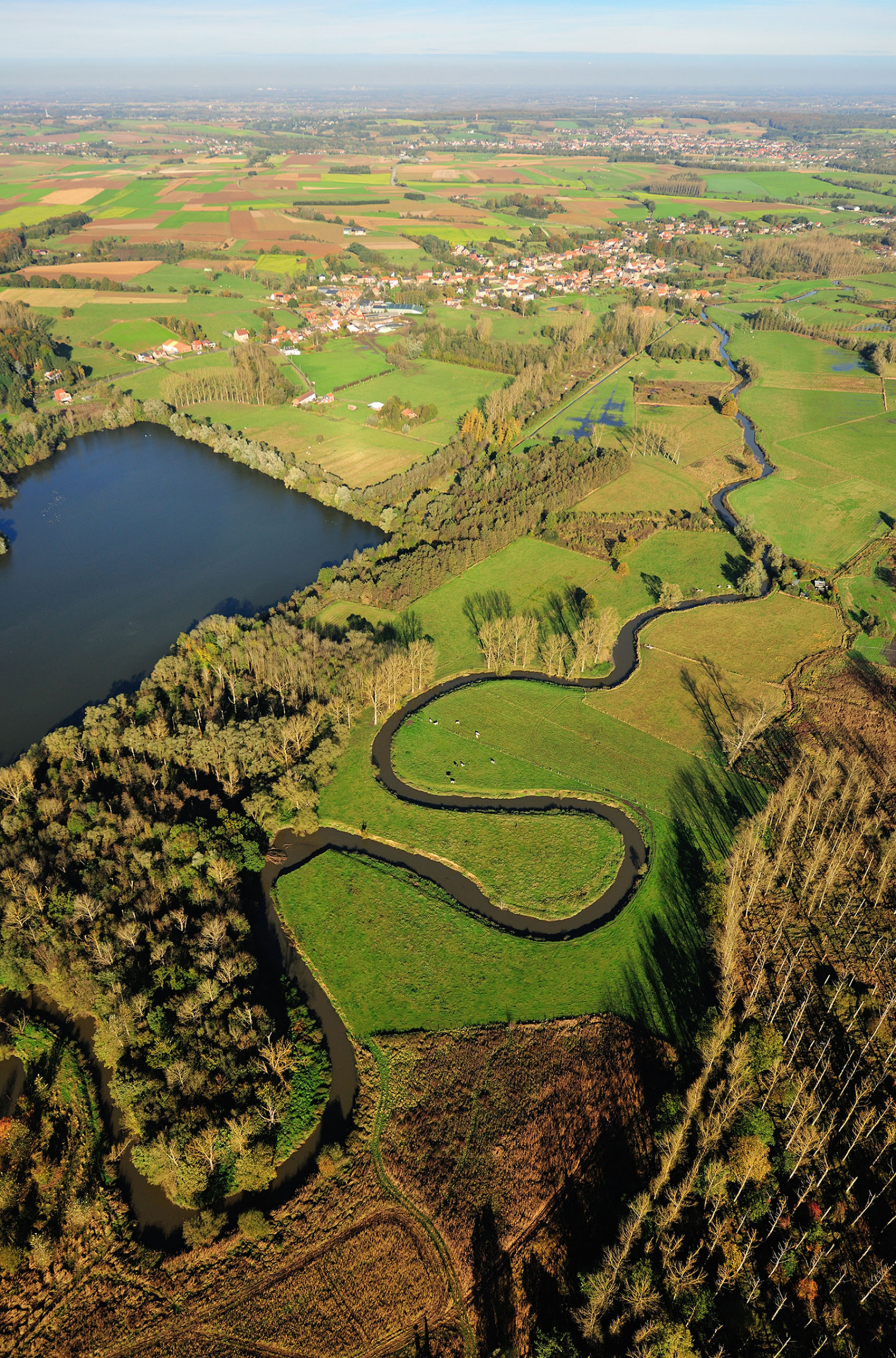
Birdseye view looking north of the Dijle valley (Doode Bemde), central Belgium. Photo: Yves Adams/Vildaphoto

Until the 1980’s, the valley was mainly used for agricultural purposes (such as hay making, livestock grazing and poplar tree cultivation) and leisure houses. As the area harbours a high level of biodiversity, including rare species and habitats, the entire valley and surroundings was designated as a “nature area” in 1975 by the Regional Destination Plan. In 1979 the complete valley floor of the Dijle was designated European Bird Directive area, and in 1992 80% of the surface was also designated European Habitat Directive area. The protected habitats are (followed by their Natura 2000 code, European Commission 2013): eutrophic lakes with Magnopotamion vegetation (3150), Alopecurion grasslands (6510), Filipendulion tall herb vegetation (6430), mires (7140) and Alion forests/alder carr (91E0). The valley is nowadays covered with grasslands, derelict poplar tree plantations and a limited surface of natural forests, ponds and some drinking water extraction sites. The southern part is called ‘Doode Bemde’ and is a nature reserve managed by a local nature conservation NGO (Vrienden van Heverleebos en Meerdaalwoud), while the northern part is a nature reserve called ‘Vijvers van Oud-Heverlee’, managed by the Agency of Nature and Forestry (ANB). The management of the river itself is under the responsibility of the Flanders Environment Agency (VMM). Residential areas are concentrated along the valley sides, while cropland is situated at the fertile western plateau (Fig. 2).

### Flood protection of Leuven: A short historical record

In early medieval times, due to large scale deforestation, the hydrological regime of the river shifted from a fairly constant base flow river into an alluvial (i.e. frequently flooding) river. Heavy rain storms erode soil with associated nutrients, mainly from the western plateaus and slopes, and runoff water transports it towards the valley bottom. Peak discharges brought huge amounts of erosion sediments into the valley. This allowed agricultural practices in the valley, but also increased downstream flood risks. Until 1990 the river water quality used to be very poor with extremely elevated nutrient levels and a ditto load of heavy metals (e.g. Cu, Pb). A decennium of sewer constructions and connecting local sewers to sewage water treatment plants increased the water quality significantly. Similar historical evolutions have occurred all over Western Europe (Huybrechts 1989; Notebaert 2009).

The process of decision-making regarding flood protection of Leuven is described by Craps et al. (2005), and complemented with information from interviews with two protagonists.

**Fig. 3:**
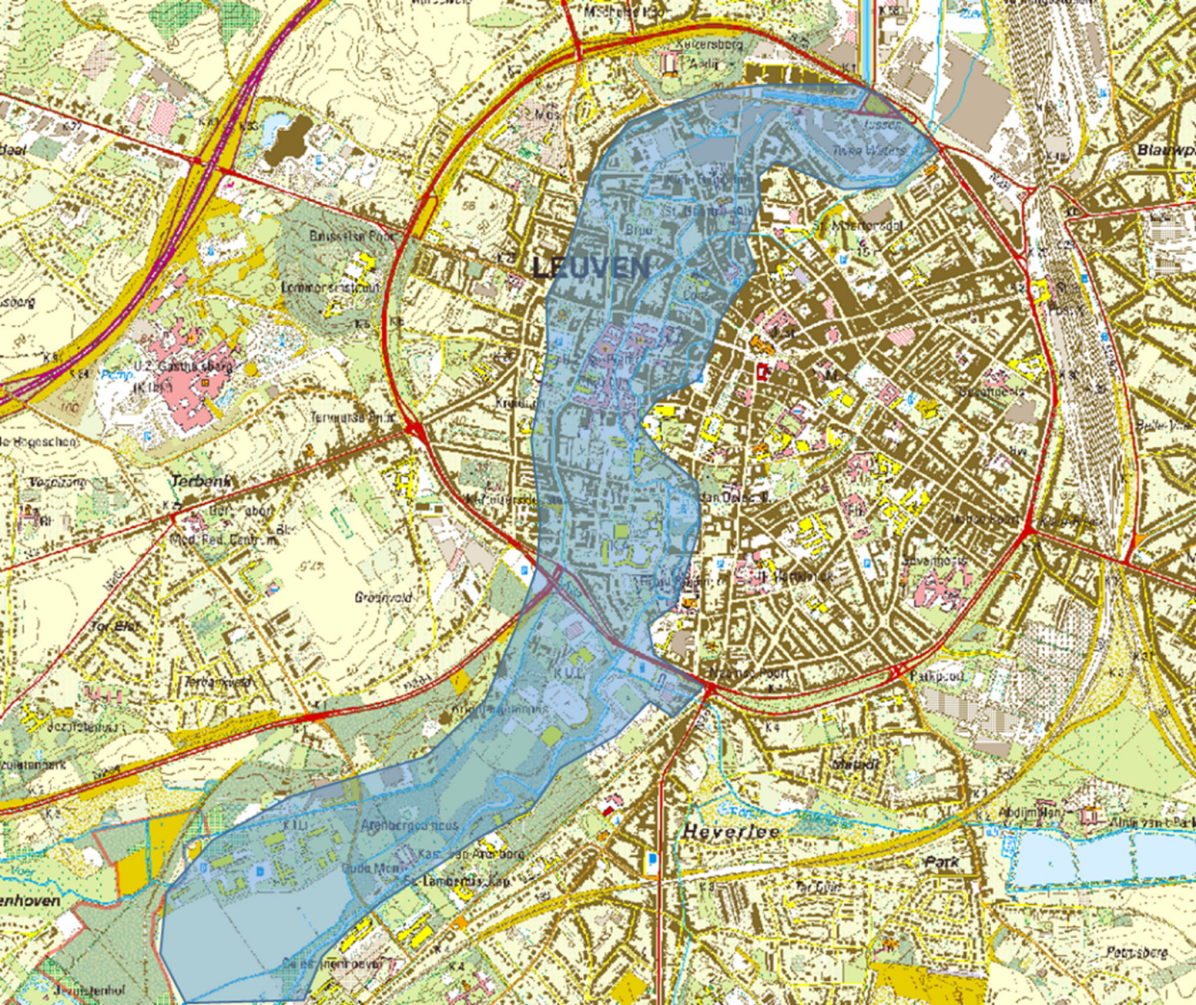
Extent of the floodable area in the historic city centre of Leuven and the university campus south of the city (after Belgroma 1990)

After the second world war, the urban sprawl of the city of Leuven expanded in the natural floodplain of the Dijle. As a result, flood risk increased sharply. Among the assets at risk are 125 ha of urban area (one third of the buildings in the historic city of Leuven), a university campus, a hospital, major roads and other critical infrastructure (Fig. 3). During the 1970’s and 80’s, public water administrations were under rising pressure to come up with a plan to avoid these growing economic and social risks. The interventions should protect Leuven against a one in hundred years’ event. As the flow capacity of the Dijle river within the city centre was presumed to be limited to 21 m^3^/s, the excess volume of a unit hydrograph of a T100 event (1 200 000 m^3^) had to be stored upstream in the Dijle valley. Discharges more than 25 m^3^/s occur every 2–10 years (Belgroma 1990, 1996). At that time, flood defence designs were based on static calculations with design storms and ignored natural flood conditions. Based on these model results, water managers formulated a plan to install storm basins that can temporarily store the excess volume of river water.

During the 1980’s a nature conservation NGO started to create a nature reserve in the valley, and considered these plans as a threat. Government agencies at that time did not recognise these environmentalists as a legitimate party and ignored them. The environmentalists tried to increase public awareness by contacting newspapers, radio and television programs, organizing guided walks and public hearings, and by motivating farmers and recreationists to submit complaints. In this way, they were able to increase the pressure on the decision makers. In 1990 the environmentalists started to incorporate flood prevention aspects in their nature conservation plans, and tried to convince the administrations that a different, nature-oriented flood control approach was an equally valuable solution. In 1993, due to the newly adopted legislation on environmental impact assessment, the administration was required to look for an alternative option that was less damaging for the environment. In the same year, hydrodynamic models were used for the first time in Flanders, which could also assess floods occurring in natural floodplains. The opposing parties gradually reached a consensus due to a number of reasons: the new European natural environment safeguarding directives, fact-based discussions based on new models, the fact that the NGO became recognized as a legitimate discussion partner, and active lobbying of the NGO with the responsible decision makers. After a long decision-making process (lasting about 25 years), a conclusion was reached in 2000, when the technical solution (based on the construction of storm basins) was abandoned in favour of a ‘nature-based solution’ (based on restoration of the alluvial floodplain, plus one emergency storm basin). The implementation of the NBS took 5 years (2000–2005).

### Comparison of two flood risk management solutions

In the 1990s, two approaches for flood damage protection were considered in order to guarantee the safety against floods, with a return period of once in 100 years. The most fundamental difference between the technical solution and the NBS is the strategy to store the excess peak discharge water volume.

#### Technical solution: Storm basins approach

In the technical solution downstream flooding is avoided by storing the excess flood water at peak discharges in storm basins, where the water is retained for a couple of days before being gradually released back into the river. This technical solution would require new measures on top of existing, recurrent interventions (Belgroma 1990, 1996):

- Installation of two storm basins (Egenhoven and Neerijse) and a third emergency storm basin (Korbeek-Dijle) (Fig. 1). The most downstream storm basin would be activated first, and was estimated to be filled with a return period of once every 3 years. If its full capacity would be reached, then the most upstream storm basin would be activated, with an estimated frequency of once every 15 to 20 years. The emergency (middle) storm basin would be activated only when the retaining capacity of the other two basins would be insufficient, and estimated to fill once in every 40 to 50 years (Table 1). The basins would be surrounded by 14 km of dikes, while the in- and outflow would be regulated by concrete infrastructure, including automatic gated weirs and pumping stations. An area of 10 ha in the lowest part of the Doode Bemde would serve as a sediment trap.

**Table 1:** Technical characteristics of the two alternative floodwater solutions to protect the city of Leuven (Belgroma 1996; flooded floodplain area mapped in situ by INBO during 1990-1994)

Location to store flood water	Estimated flooding frequency	Water storage capacity (for a T100 event) (m^3^)	Total surface area (ha)	Dikes length (m)
*Technical solution: Storm basins approach*				
Storm basin Egenhoven	Once/3 years	887 000	72	5950
Storm basin Neerijse	Once/15-20 years	1 500 000	136	3250
Emergency storm basin Korbeek-Dijle	Once/40-50 years	1 000 000	125	4875
*Nature-based solution: Restoration of the alluvial floodplain*				
Storm basin Egenhoven		800 000	74	1500
Alluvial floodplain south of E40		3 500 000	898	0
- Maintenance works on the river channel would be continued, to avoid turbulent flow and river bank erosion, and consequent financial claims from land owners. Since this smoothening of the river channel reduced the channel roughness, peak discharge water volumes were transported very fast through the river channel, completely surpassing the alluvial floodplain, and thus increasing peak discharges and the consequent downstream flood risks.
- Regular maintenance of drainage channels would be continued and a siphon would be maintained. These interventions aimed to lower the groundwater level for the predominantly agricultural use of the floodplain, especially in areas with upward groundwater seepage flow.

#### Nature-based solution (NBS): Restoration of the alluvial floodplain

The main idea of this strategy is to restore a more ‘natural’ flooding regime in the floodplain and to restore the alluvial floodplain ecosystem (Fig. 1). This NBS requires four main (non)-interventions:

- Making use of the storage capacity of the entire natural floodplain: The microtopography of the alluvial floodplain is a crucial feature for this solution. Due to regular floodings since the early medieval times, the river has formed natural elevated banks and lower lying floodplain depressions. When the river channel reaches bank-full (and higher) discharges, the water will overflow the river banks and inundate the floodplain depressions along the entire length of the river simultaneously (± 12 km). Consequently, flood water including the sediment load will spread out over a large part of the natural floodplain.
- ‘Zero management’ of the river and its banks: This means that the watercourse through the Doode Bemde is no longer cleared from fallen trees, and the river banks are no longer mown. Fallen trees in the river increase the roughness of the watercourse. Consequently, bank-full discharge is reached increasingly sooner (or at lower discharges) and spontaneous floods in the floodplain are induced. Zero management of the river banks of the Dijle started in 1991, but took about a decade before the woody vegetation had the required beneficial impact on increased river bank roughness.
- Reconnecting the Leigracht to the IJse (i.e. tributary of the Dijle) by removing a siphon (2002) inevitably led to higher groundwater levels, which is favourable for the restoration of groundwater-dependent vegetation types. In addition, this also helped to restore the relation between the river and its surrounding floodplain (La Rivière 2006).
- Minimum infrastructure works (only on a limited number of locations at the fringes of the floodplain). Eventually, it was decided to include the Egenhoven storm basin as an ultimate emergency protection for floods in Leuven in the event of extreme rainfall south of Leuven (completed in 2005).

If the river roughness is high enough and the storage capacity of the natural floodplain depressions is large enough to store the excess stormwater volume, flood damage in the city of Leuven can be avoided. In other words, the flooding of the floodplain should start at a discharge level which is lower than the flow transport capacity of the river through the city centre.

## Materials and methods

### Comparative social cost–benefit analysis (cSCBA)

**Fig. 4:**
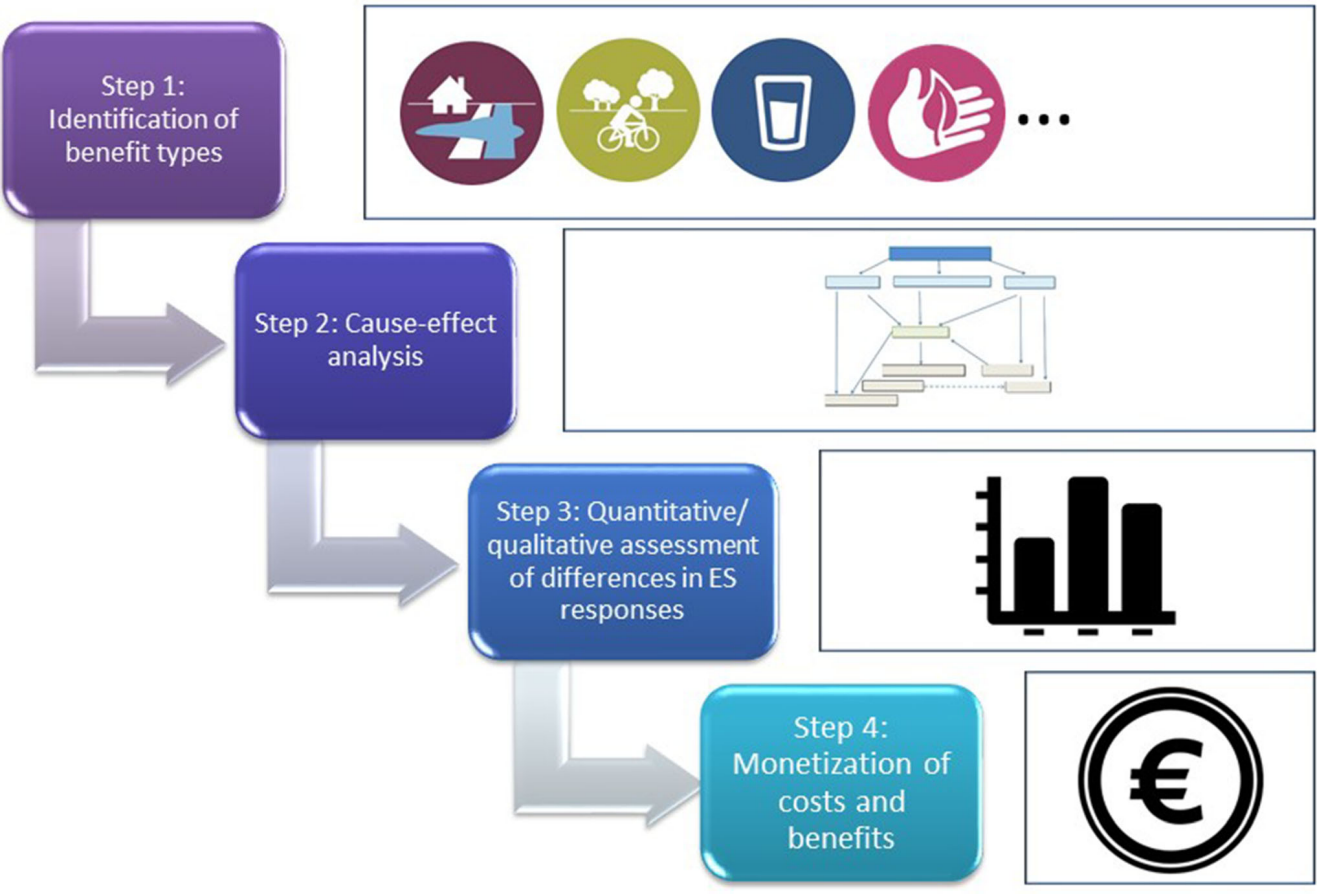
Different steps of the comparative social cost–benefit analysis (cSCBA) to assess the differences in the costs and benefits between the technical solution and the alternative NBS for flood control

The method we selected to compare between a nature-based and a technical solution is a social cost–benefit analysis (SCBA) (OECD 2018). This is a project appraisal approach that enables to inform policy decision-making, by mapping all the costs and benefits for all parties concerned and weighing them against each other. In a classical SCBA, all possible costs and all possible benefits for both solutions are examined. Benefits represent an increase in human welfare or wellbeing, while costs a decrease in human welfare or wellbeing. In this study, we opted for a comparative SCBA where only those costs and benefits are considered for which differences between the two solutions were expected (Demeyer and Turkelboom 2013b). However, comparison between these two solutions was not always evident. The current situation was considered as a representation for the NBS, for which we could use actual measured data. As the technical solution is a hypothetical approach, we therefore had to use modelled data, data of 20 years ago and expert estimates. To address the uncertainties of this approach, we often calculated low and high estimates. As some assumptions needed to be made, all the results were discussed and validated by an interdisciplinary expert group during 3 meetings. This group included experts from the Flemish Environment Agency (water management), Agency of Nature and Forestry (nature & biodiversity), Department of Environment (policy) and the Institute of Nature and Forest Research (ecohydrology). Our approach entailed 4 sequential steps (Fig. 4).

### Step 1: Identification of benefit types

In order to identify the local-relevant benefits (ecosystem services (ES) and/or biodiversity), we interviewed 10 key informants who had a helicopter view (during 2013). To ensure a diversity of opinions, we looked for people with opposing views concerning the study area. The interviewees included representatives from a water management organization, drinking water company, nature protection agency, land development organization, service organization for municipalities, local nature NGO, hunters’ association, forest owners’ association, a local farmer and a kayak renting company owner. Respondents were asked to rank pictures of benefits according to their importance for the study area (potential scores: 3 (very important), 2 (medium important), 1 (bit important), 0 (neutral), − 1 (not desired)). The identified benefits were validated by the expert group, who also added some extra ES.

### Step 2: Cause-effect analysis

As we wanted to apply a comparative SCBA, it was necessary to withhold only those benefits that respond differently to the two proposed flood management solutions. The tool to justify this selection was a cause-effect flowchart, which illustrates how the two flood management solutions are triggering different responses of ES and biodiversity. This chart was designed based on discussions with the expert group, and it was built in an iterative way.

### Step 3: Quantitative/qualitative assessment of differential impacts

The difference in ES responses between both solutions were quantified based on the formulas used in the ‘Nature Value Explorer’ (NVE). The NVE is an online tool for assessing the impact of land use changes on ES in quantitative and monetary terms, based on the best available empirical knowledge in Flanders (Liekens et al. 2013). For those impacts for which NVE formulas were lacking, we referred to available empirical data, literature and expert judgement.

### Step 4: Monetization of costs and benefits

Monetization is the conversion of the quantified effects into monetary terms. The investment and management costs of the two approaches were extracted from an EIA report (Arcadis 2012). To assess the differential impacts on ES, we used the NVE. When the obtained monetary values of cSCBA were prone to uncertainty, a high and low estimate were calculated. Another complication was that cost and benefits appear in different periods over time. To compare all present and future costs and benefits, they were converted to present time value (i.e. 2013). For this purpose, a discount rate of 4% was used (as proposed in the NVE).

**Fig. 5:**
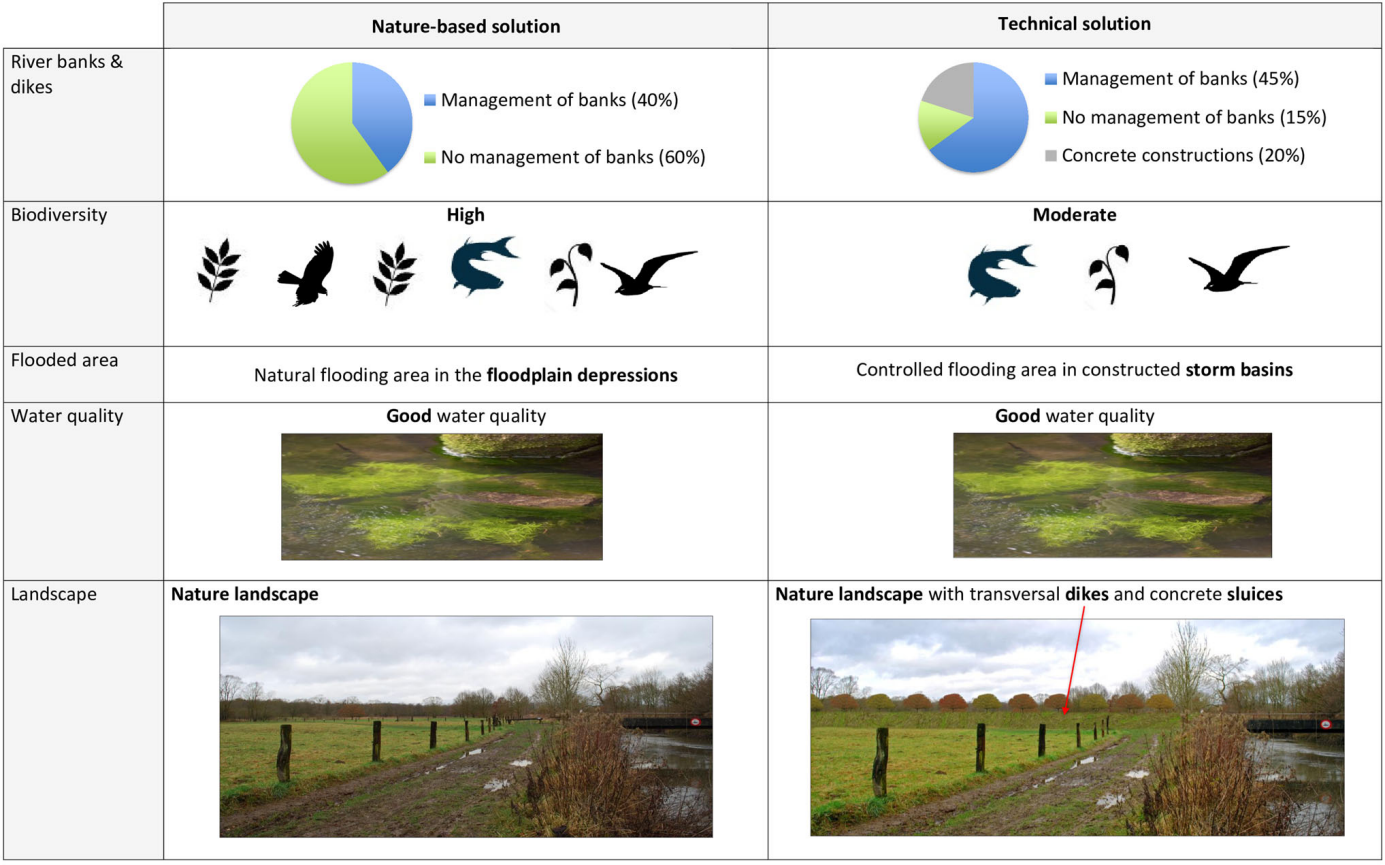
Example of a choice card, with two solutions (columns) and five features (rows)

As the NVE was not specific enough to determine the recreational value for both solutions, an online survey was drawn to elicit preferences of respondents between the technical solution and the NBS (Supplementary Material S1). For this purpose, we used the contingent valuation method (Mitchell and Carson 1989; Hanley et al. 2001). Respondents were presented with two choice cards with each two flood management approaches: one card contained the NBS and the technical solution, the other the NBS and the 1995 situation (Fig. 5). To avoid overload and possible drop-out of respondents, a third card (comparing 1995 situation with the technical solution) was not presented. Five features or attributes were used to describe the approaches: characteristics of river banks (% of banks with and without management, and % concrete dikes), biodiversity (high, medium, low), flooded area (natural flooding area, flooding in a storm basin with concrete dikes, no flooding area), water quality (good, medium, poor), landscape (natural landscape; natural landscape with presence of dikes; agricultural landscape with meadows and poplar trees). In the study we assumed that access to the area would be equal for the three management approaches. Next, respondents were asked about their willingness to pay for their preferred approach using double bounded dichotomous choice questions as the elicitation method (Perman et al. 2011). This amount would then have to be paid to a government body (e.g. as a kind of tax) to realize the chosen approach. Twelve starting bids that ranged from 5 euro to 60 euro were used for the first contingent valuation question and were randomized over the sample. In the follow-up question the amount was—depending on the answer on the first question—increased or decreased with 5 euros. At the end, we asked some questions about the socio-demographic background of the respondent. In total 332 people completed the survey, of whom 89% were familiar with the area (Coucke 2013).

## Results

In this section we discuss the differences between the costs and benefits of the technical solution and the NBS.

### Identification of benefit types (Step 1)

The interviewed stakeholders identified the following functions as most important for the study area (in declining order): 1) habitat for (typical) animals and plants, 2) protection against floods, 3) clean water, 4) recreation (walking and cycling), and 5) experience of the landscape (i.e. aesthetic value, therapeutic effect, historical landscape). In addition, another 15 ES were positively evaluated. The expert group added two ES that were not considered by local stakeholders: carbon sequestration and air quality improvement. In total 20+ important benefits were identified.

### Cause-effect analysis (Step 2)

**Fig. 6:**
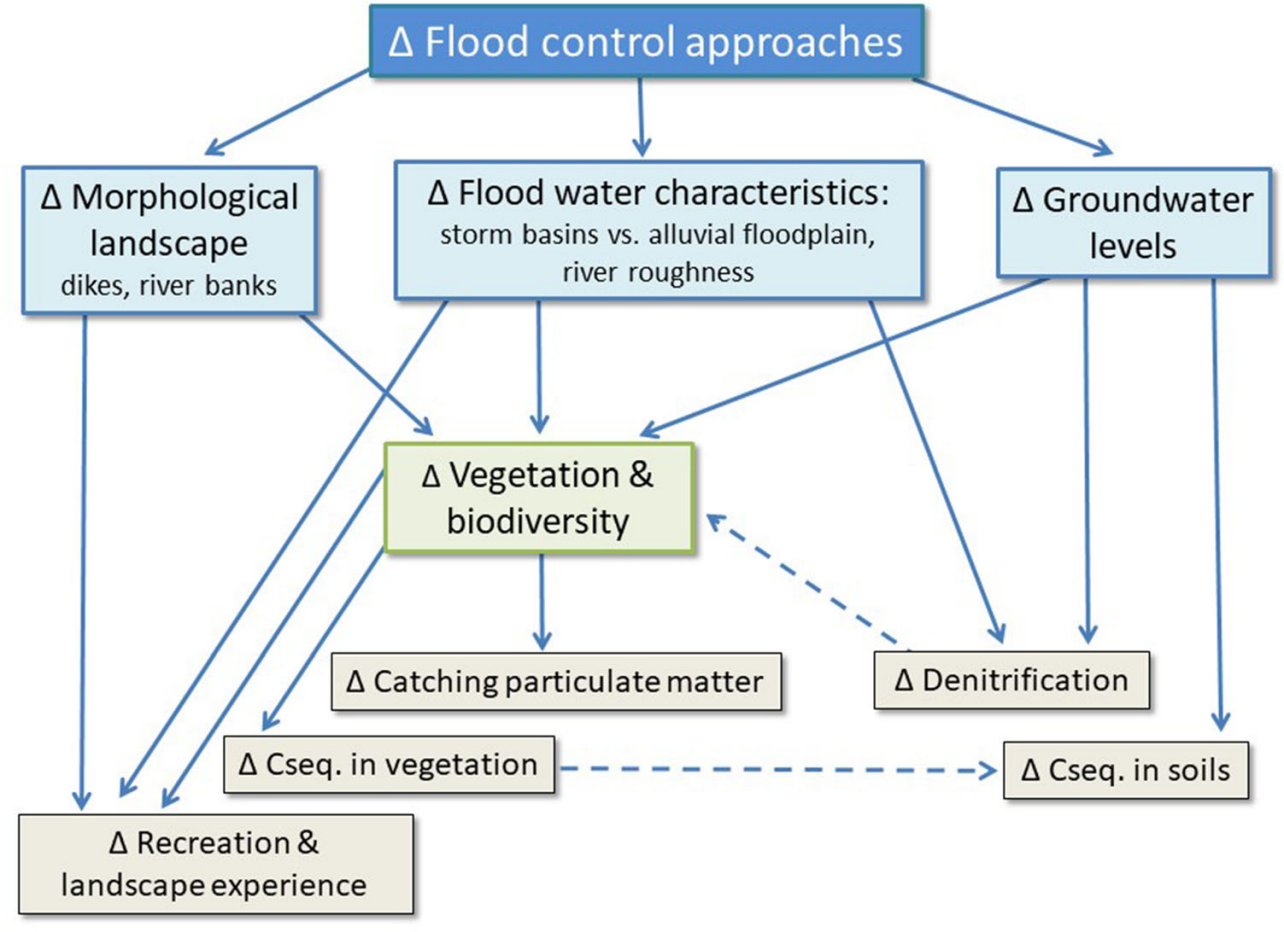
Cause-effect flowchart showing the differential impacts of flood control approaches on ES delivery and biodiversity in the Dijle valley (blue blocks: controlling factors; green block: biodiversity; grey blocks: ES)

However, not all these benefits needed to be assessed for this analysis, as we are only interested in those benefits which respond distinctively to the two flood management approaches. Via the cause-effect analysis, we found that there are three major intermediate controlling factors which respond differently to each management approach (Fig. 6):

- The morphological landscape of the valley floor is mainly affected by the dikes and sluices and the management of the river banks.
- Flood water characteristics: As in the technical solution the flood water is stored in a limited area, the flood water will be deeper (89, 10, 0 cm for T10 storms for the respective storm basins), the retention time shorter (2, 1, 0 days for T10 storms), and sediments will be mainly concentrated in the storm basins. In the NBS, the excess water volume is spread over a larger surface, resulting in a lower depth of the flood water (average 16 cm), a longer retention time (median: 2.5 days, range: 1–16, measured in Neerijse floodplains 2008–2012), and spread of the sediment over a larger area. This reduces the thickness of the sediment layer to millimetres instead of centimetres compared with the storm basin sedimentation rates (Belgroma 1996; De Becker and De Bie 2013).
- Groundwater levels: In the technical solution, the drainage canals remain active and the water is drained faster via the cleared river, resulting in an overall lower groundwater level. The removal of the siphon in the NBS resulted in an increase of the groundwater level (average 9–17 cm higher for resp. high and low groundwater levels) (De Wilde et al. 2001; De Becker and De Bie 2013).

These controlling factors influence five ES, vegetation and biodiversity in distinct ways (Fig. 6).

### Impacts of flood management approaches on ES and vegetation (Step 3)

Only those benefits which respond differently for the two solutions are assessed (Table 2):

**Table 2:** Comparative SCBA for floodwater management solutions in the Dijle. Positive values mean that NBS provides more ES (and euros) compared to the technical solution and vice versa

Additional benefits of nature development approach	Qualitative/quantitative assessment			Economic value (×1000€) for 30-year period*	
	Units	Low estimate	High estimate	Low estimate	High estimate
Denitrification	Ton N removal/ year	+ 11.3		+ 951	+ 14 075
Carbon sequestration in soil	Ton C sequestration/ year	+ 542	+ 554	+ 2011	+ 2328
Biodiversity		Increase			
Carbon sequestration in vegetation	Ton C sequestration/ year	− 87		− 346	
Air quality improvement	Ton particulate matter removal/ year	+ 0.28	+ 1.05	+ 271	+ 1020
Recreation & landscape experience				+ 29 044	+ 83 390
Avoided costs (Table 3)				+ 2470	+ 2540
**Total**				**+ 34 401**	**+ 103 007**

*: 4% discount rate over a time horizon of 30 years

**Flood control**: Modelling studies using the first generation hydrodynamic computer models showed that the storm basin and nature development approaches are equally capable of protecting Leuven against floods that occur once every 100 years (1 200 000 m^3^, Belgroma 1996). As both approaches offer similar protection against floods, this impact was not included in the cSCBA. Recent calculations of high-end climate change scenarios reveal that in the case of an extreme event, additional measures will have to be taken in the future to prevent increasing flood risks in the lower parts of the city (VMM 2014).

**Water quality improvement via denitrification:** Denitrification is the conversion of nitrate (NO3^-^) to nitrogen (N_2_) by bacteria, which is released into the air. This contributes to improved water quality. An average nitrogen concentration of 5,7 mg N/l in river water and 2,5 mg N/l for ground water were used (De Wilde et al. 2001). Separate denitrification estimations were made for terrestrial ecosystems, areas with temporary flooding and running water. The NBS scored better as denitrification efficiency is higher in wet, terrestrial ecosystems (due to higher groundwater level: +20 cm), in flooded areas (due to larger flooded surface) and in running water (due to 10 cm higher water level and hence greater water volume). As a result, an extra 320 tons nitrogen is denitrified over a period of 30 years in the NBS.

**Carbon sequestration in soil**: The amount of C that is stored depends on land use and hydrology: wetting leads to a greater C stock, while drainage leads to less C storage in soils. The NBS results in an additional 542/554 tons C per year in the soil, due to higher groundwater levels (+20 cm) and a greater area of swamps and reed (+67/78 ha), compared to the technical solution. This amount is possibly an overestimation, as we had to rely on groundwater levels measured in one particular floodplain, where measures were taken to increase the groundwater level.

**Vegetation** and **biodiversity** is to a large extent determined by the management of the river banks, the flood water characteristics and the groundwater level (Fig. 6). Inside the storm basins of the technical solution, a high water column of standing water with the complete sediment load, would cover the vegetation with inches’ thick sediment cover. Since the Dijle was at that time heavily polluted and richly loaded with nutrients, the resulting vegetation in these storm basins would be of very limited ecological and aesthetic value. Outside the basins, the vegetation would be mainly influenced by the lower water table.

In the NBS, the increased groundwater level is the main source of shifts in vegetation types: Filipendulion tall herb vegetation (Natura2000 code 6430, e.g. meadowsweet) and Alopecurion grasslands (6510, e.g. meadow foxtail) increased in acreage (approx. +73 ha) predominantely at the expense (− 55 ha) of drier grassland types (crested dog’s-tail (*Cynosurion cristati)* and tall oat grass (*Arrhenaterion)* grasslands) (Demeyer and Turkelboom 2013a; based on Belgroma 1996; De Wilde et al. 2001; De Nocker et al. 2006; De Becker and De Bie 2013). A survey indicated that the changes in the water regime between 1990s and 2019 have also led to an increase in species diversity: typical vegetation types who were present in the Dijle valley before the interventions have remained but shifted to higher grounds (e.g. tall sedge swamps (with *Carex acuta & acutiformis)* and marsh marigold (*Calthion palustris*) grasslands), while vegetation types of wetter conditions have emerged, with associated invertebrate and vertebrate species (De Becker and De Bie 2013; De Becker 2020). In addition, as the river channel in the NBS can move more freely, small beaches, eroding banks and steep, vegetation-free banks are formed. The latter are an ideal breeding place for e.g. kingfisher (*Alcedo atthis*). In the river itself, a natural pool-riffle pattern developed, which is favourable for aquatic biodiversity (La Rivière 2006). This is in line with other studies showing a greater species diversity in areas where a complete gradient of flood characteristics is present, compared to a river with technical structures and abrupt hydrological conditions (de Nooij et al. 2006; Pettifer and Kay 2011).

**Carbon sequestration in vegetation:** C storage in vegetation is mainly dependent on tree growth. For both approaches, there is no difference in forest area, but trees sequester more carbon on drier soils. As the technical solution results in drier soils (groundwater table 20 cm lower), an additional 87 tons of carbon per year are stored compared to the NBS.

**Air quality:** Vegetation has a positive impact on air quality, as leaves capture particulate matter. Particulate matter is responsible for many of the diseases caused by environmental pollution. The forest areas are equal in both solutions. But as the NBS has more swamps and reed areas (+67/78 ha), there is an additional capture between 279 and 1051 kg particulate matter per year.

**Recreation and landscape experience:** The majority of the respondents (77%) preferred the NBS, while 23% preferred the technical solution. An impact that was not explicitly assessed was the unique value of the valley in Flanders, as it is one of the few remaining natural alluvial systems in which a freely meandering river is allowed to flood its natural floodplain. This would probably represent an additional benefit for the NBS.

### Monetization of costs and benefits (Step 4)

The costs can be divided into two categories: investment costs (for construction of infrastructure and equipment) and maintenance costs (maintenance of infrastructure and nature management). A summary of the differences in costs between both approaches is summarized in Table 3: the technical solution requires a one-time extra cost between 2.65 and 2.72 million €, while the annual running cost is 10 000 € lower. Overall, the technical solution (investment and maintenance costs) would cost 2.4 to 2.5 million € extra over a 30-year period compared to the NBS.

**Table 3:** Summary of differences in investment and maintenance costs between the technical and nature-based solutions (Demeyer and Turkelboom 2013a, based on Arcadis 2012, and estimates of VMM and Piet De Becker)

Additional costs for technical solution	× 1000€	Additional costs for NBS	× 1000€
*Investment costs*			
Storm basin Neerijse	1300	Removal of siphon of Leigracht under the Ijse river	30–50
Storm basin Korbeek-Dijle	1300		
Weirs for real-time control for Neerijse & Korbeek-Dijle storm basins	50		
Dikes around drinking water abstraction area	50–100		
*Maintenance and management costs per year*			
Pruning of vegetation along the river banks, and dredging of Dijle river channel	88	Additional cost for nature management (esp. mowing of grassland and removal)	108
Operational cost of storm basins	10		
**TOTAL extra cost technical approach (×1000€)**		+2650 to 2720 (investment) - 180* (maintenance)	

*For a time horizon of 30 years at a discount rate of 4%

Monetary benefits of ES are calculated with NVE (Table 2). Regarding recreation, the respondents who preferred the NBS were willing to pay (WTP) on average 8 €/month/household (p<0.05) to retain the NBS (with a 95% confidence interval from 4.14 to 11.89 €/month/household). Age, income and being unemployed had a significant positive effect on the WTP, while proximity of nature had a significant negative effect on the WTP. As a large part of the survey respondents (44%) were donating money to nature-related organizations and as there was a significant correlation between this characteristic and the degree of WTP, this number is probably an overestimation (Coucke, 2013). On the other hand, such a high rating is not exceptional, considering that the nature reserve is surrounded by densely populated areas. Similar values for recreation were also found in previous SCBAs in Flanders (De Nocker et al. 2005; De Nocker et al. 2011). If we multiply the obtained WTP amount by 12 (to obtain a yearly amount) and the number of households of the municipalities that provide most recreational users for the study area (6 municipalities with 32 500 inhabitants), an average willingness to pay of 3 120 000 €/year was obtained (with a 95% confidence interval from 1 615 000 to 4 637 000 €/year) (Coucke, 2013).

If we sum the differences of costs and benefits affected by both solutions, the NBS delivers an additional value between 32 and 100 million € over a period of 30 years, compared to the technical approach (Table 2). The used discount rate in a SCBA is always subject of debate (Gowdy et al. 2010; Perman et al. 2011). A positive discount rate in fact implies that the present value of future costs and benefits is given less weight than the current values. The discount rate can be set lower than 4%, or even a negative rate could be used. A negative discount rate is based on the assumption that nature in future will be more valuable than today. This could be argued based on the fact that there will be an increasing population and a higher demand for ES in the future. When we would have taken a lower discount rate, the additional benefits for the NBS would have been even higher, as compared to the technical solution. For example, if we use a discount rate of 0%, the added value of the NBS would amount between 54 and 147 million € over 30 years.

## Discussion

A NBS for flood control with restoration of the alluvial floodplain can be considered a valid alternative for a technical solution. The major advantages are the potential to provide the same flood protection for less costs, but with additional co-benefits for society and an increase in biodiversity. In addition, the nature-based solution is a “no-regret solution”, as it is able to tackle future challenges, such as climate change. If the authorities would have chosen for the technical solution—fully relying on construction works—any alteration would lead to excessive costs and additional threats to nature values in the Dijle valley. Based on these results, we can conclude that policy-makers made in the year 2000 the right choice to opt for the NBS for the Dijle valley. The ‘Dijle case’ can be considered as an early positive example of integrated basin management, in the spirit of the European Water Framework Directive (2000/60/EC) and the EU Floods directive (2007/60/EC).

On the other hand, it is important to note that the results of a cSCBA for different flood control solutions is highly context-dependent. In the case of the Dijle valley, the floodplain has a protected status, and housing was never very important in the valley due to its waterlogged soils. Consequently, the opportunity costs for housing and agriculture were close to zero. In a valley which is intensively farmed and/or where there are residential areas, the opportunity costs for a NBS would be much higher. When available space is a crucial issue or when opportunity costs are high, a technical solution might be more suitable, although it still needs to be considered that technical solutions will provide less ES and/or biodiversity.

A possible consequence of the advantages of such NBS could be that the ever smaller remaining fragments of wetlands are being considered to provide flood protection services. As these areas are usually under nature conservation legislation, this can entail some risks. Nature conservation and flood damage protection are compatible when some key elements are taken into account: flood water has to be of sufficiently good quality and the amount of excess (flood) water has to be stored on a large as possible surface (De Becker & De Bie 2013). In other words, the entire natural floodplain should be used in order to reduce the flood frequency, duration, water depth as well as sediment load per unit surface to a minimum. In this way, the negative impact on biodiversity will be minimized. All those elements were taken into account in the flood damage control discussion for the Dijle. Considering the urbanistic developments in the Dijle river catchment, this approach was the best option to achieve both the Natura 2000 goals and the required flood damage protection. Therefore, this type of NBS fits well in the definition for NBS Type 1: no or minimal intervention in the ecosystem, with the objectives of maintaining or improving the delivery of a range of ES both inside and outside of these preserved ecosystems (Eggermont et al. 2015).

Despite the limitations we encountered, we suggest that a comparative social cost–benefit analysis, supported by a cause-effect analysis, based on data from models and local knowledge, and validated by an expert group, is a pragmatic approach to make informed decisions. However, a comparison between a NBS and a technical solution is not always a grey vs. green comparison. A NBS may include a number of technical measures (e.g. the NBS for the Dijle valley also included one emergency storm basin). In contrast, technical solutions can also include green elements.

A final interesting observation of this case is that the debate took 25 years, while the implementation only required 5 years. A clear environmental policy framework, availability of appropriate flood risk models, and an active involvement of all stakeholders in the early phase of the debate would have probably reduced the length of the debate period.

## Conclusion

As flood damage protection issues are increasingly important all over Europe, NBS are often presented as a valid alternative for technical solutions. To make a comprehensive comparison, it is important to not only focus on the level of flood damage protection and investment and maintenance costs, but also to consider all other impacts on ecosystem services and biodiversity. From a successfully implemented NBS in the Dijle valley in Belgium, we can—despite some uncertainties—confidently state that the NBS provides the required flood security, for fewer costs and with more ecosystem services benefits and biodiversity, compared to the technical solution. The highest additional values are realized via recreation, denitrification, and biodiversity. Recreation comes out as the most valuable ES provided by the NBS (83–91% of the total extra value of the NBS). Only carbon sequestration in vegetation scored better in the technical solution. Reconnecting rivers with their floodplains is, therefore, a valuable policy option when coping with flood risks. However, the business case for working with NBS depend a lot on the spatial and socio-ecological context: the opportunity for a NBS increases when there is sufficient space to retain flood water, when flood water is of sufficient good quality, and when there are only limited economic activities and/or residential areas in the floodplain.

## Supplementary Information

Below is the link to the electronic supplementary material.

Supplementary file1 (PDF 513 KB)

## References

[CR1] Arcadis (2012). Development of a model for the technical costs of nature development and management activities.

[CR2] Albert C, Schröter B, Haase D, Brillinger M, Henze J, Herrmann S, Gottwald S, Guerrero P (2019). Addressing societal challenges through nature-based solutions: How can landscape planning and governance research contribute?. Landscape and Urban Planning.

[CR3] Belgroma. 1990. Complementary study of flood protection on the river Dijle upstream of the city of Leuven. Belgium (in Dutch).

[CR4] Belgroma. 1996. Study of flood protection on the river Dijle upstream of Leuven including the nature development scenario after calibration and including groundwater production installations. Belgium (in Dutch).

[CR5] Buijse AD, Coops H, Staras M, Jans LH, Van Geest GJ, Grift RE, Ibelings BW, Oosterberg W (2002). Restoration strategies for river floodplains along large lowland rivers in Europe. Freshwater Biology.

[CR6] Coucke L. 2013. The value of recreation in the Dijle Valley. A comparison of different water management scenarios. MSc Thesis. K.U.Leuven, Leuven, Belgium.

[CR7] Craps M, Van Rossen E, Prins S, Taillieu T, Bouwen R, Dewulf A, Wildemeerch D, Stroobants V, Bron M (2005). Relational practices to make social learning happen: A case study in water and nature management. Active Citizenship and Multiple Identities in Europe.

[CR8] De Becker P. 2020. Ecohydrological system descriptions for nature reserves in Flanders in the context of PAS. Research Institute for Nature and Forest, INBO.R.2020.12, Brussels, Belgium (in Dutch, English summary).

[CR9] De Becker P., and E. De Bie. 2013. Gathering basic knowledge and developing an assessment framework for the ecological impact analysis of floods. Research Institute for Nature and Forest, INBO.R.2013.6, Brussels, Belgium (in Dutch).

[CR10] De Nocker L, Liekens I, Broeckx S (2005). Wetlands in the estuary of the river Scheldt. An appraisal of the costs and benefits.

[CR11] De Nocker L., I. Joris, L. Janssen, R. Smolders, D. Van Roy, B. Vandecasteele, L. Meiresonne, B. Van der Aa, et al. 2006. Multifunctionality and inundation areas: Scientific appraisal of the impact of water retention on nature, forest and agriculture. VITO, VITO/B/2006, Mol, Belgium (in Dutch).

[CR12] De Nocker L, Broeckx S, Liekens I (2011). Economic valuation of improving the ecological state of surface water using the results of the Aquaoney project.

[CR13] de Nooij RJ, Verberk WC, Lenders HJ, Leuven RS, Nienhuis P (2006). The importance of hydrodynamics for protected and endangered biodiversity of lowland rivers. Hydrobiologia.

[CR14] De Wilde M., P. De Becker, and M. Hermy. 2001. Ecohydrologial study of the valley of the river Dijle south of the city of Leuven. Research Institute for Nature and Forest, INBO.R.2001.13, Brussels, Belgium (in Dutch).

[CR15] Demeyer, R., and F. Turkelboom. 2013a. Working cost effectively with nature: ecological versus technological solutions. Research Institute for Nature and Forest, INBO.R.2013.31, Brussels, Belgium (in Dutch, English summary).

[CR50] Demeyer, R., and F. Turkelboom. 2013b. Kosteneffectief werken met natuur: Ecologische vs technologische oplossingen. Verkennende case studie: Bescherming tegen overstromingen in de Dijlevallei. https://www.vlaanderen.be/inbo/publicaties/kosteneffectief-werken-met-natuur-ecologische-vs-technologische-oplossingen.

[CR16] Eggermont H, Balian E, Azevedo JM, Beumer V, Brodin T, Claudet J, Fady B, Grube M (2015). Nature-based Solutions: New Influence for Environmental Management and Research in Europe. GAIA.

[CR17] European Commission. 2013. Interpretation manual of European union habitats. EUR 28. EC, DG Environment Nature, ENV B.3.

[CR18] European Commission. 2020. Policy Topics: Nature-Based Solutions. Retrieved 12 August, 2020, from https://ec.europa.eu/research/environment/index.cfm?Pg=nbs

[CR19] Gowdy J, Howarth RB, Tisdell C, Kumar P (2010). Discounting, ethics and options for maintaining biodiversity and ecosystem integrity. The economics of ecosystems and biodiversity: Ecological and economic foundations.

[CR20] Halbac-Cotoara-Zamfir R (2019). Nature-based solutions for flood risk management: A Romanian case study. Aktualni Zadaci Mehanizacije Poljoprivrede: Zbornik Radova: Actual Tasks on Agricultural Engineering. Proceedings. I. Kovacev and N. Bilandzija.

[CR21] Hanley N, Mourato S, Wright RE (2001). Choice modeling approaches: a superior alternative for environmental valuation?. Journal of Economic Surveys.

[CR22] Huybrechts W. 1989. Paleo hydrologic conditions in the Mark river basin during the last 15000 years. PhD Thesis. Free University Brussels, Brussels, Belgium.

[CR23] Kiedrzynska E, Kiedrzynski M, Zalewski M (2015). Sustainable floodplain management for flood prevention and water quality improvement. Natural Hazards.

[CR24] Kondolf GM, Pinto PJ (2016). The social connectivity of urban rivers. Geomorphology.

[CR25] La Rivière J (2006). The Dijle in the city of Leuven, a curse and a blessing Brussel.

[CR26] Liao K-H (2014). From flood control to flood adaptation: A case study on the Lower Green River Valley and the City of Kent in King County. Washington. Natural Hazards.

[CR27] Liekens I., K. Van Der Biest, J. Staes, L. De Nocker, J. Aertsens, and S. Broeckx. 2013. Valuation of ecosystem services: A manual Study executed for the Department of Environment, Nature and Energy. VITO, 2013/RMA/R/46, Mol, Belgium. https://www.natuurwaardeverkenner.be/

[CR28] Malmqvist B, Rundle S (2002). Threats to the running water ecosystems of the world. Environmental Conservation.

[CR29] Mitchell RC, Carson RT (1989). Using surveys to value public goods: The contingent valuation method.

[CR30] Nesshöver C, Assmuth T, Irvine KN, Rusch GM, Waylen KA, Delbaere B, Haase D, Jones-Walters L (2017). The science, policy and practice of nature-based solutions: An interdisciplinary perspective. Science of the Total Environment.

[CR31] Notebaert B. 2009. Sensitivity of river systems to human actions and climatic events across different environments: A holocene perspective. PhD thesis. K.U.Leuven, Leuven, Belgium.

[CR32] OECD. 2018. *Cost-Benefit Analysis and the Environment: Further Developments and Policy Use*. OECD Publishing, Paris, France. http://dx.doi.org/10.1787/9789264085169-en

[CR33] Opperman JJ, Galloway GE, Fargione J, Mount JF, Richter BD, Secchi S (2009). Sustainable floodplains through large-Scale Reconnection to Rivers. Science.

[CR34] Perman R, Ma Y, Common M, Maddison D, McGilvray J (2011). Natural Resource and Environmental Economics.

[CR35] Pettifer E, Kay P (2011). The effects of flood defences on riparian vegetation species richness and abundance. Water and Environment Journal.

[CR36] Posthumus H, Rouquette JR, Morris J, Gowing DJ, Hess TM (2010). A framework for the assessment of ecosystem goods and services; a case study on lowland floodplains in England. Ecological Economics.

[CR37] Schindler S, Sebesvari Z, Damm C, Euller K, Mauerhofer V, Schneidergruber A, Biró M, Essl F (2014). Multifunctionality of floodplain landscapes: relating management options to ecosystem services. Landscape Ecology.

[CR38] Tockner K, Stanford JA (2002). Riverine flood plains: present state and future trends. Environmental Conservation.

[CR39] Vandaele K, Huybrechts W, Librecht I, De Becker P, Rossaert G (2002). Evolution of the meandering of the river Dijle. Water.

[CR40] VMM (2014). Support for the flood risk management plan for unnavigable watercourses.

[CR41] Walz U, Richter B, Grunewald K (2019). Indicators on the ecosystem service “regulation service of floodplains”. Ecological Indicators.

[CR42] Wantzen KM, Ballouche A, Longuet I, Bao I, Bocoum H, Cisse L, Chauhan M, Girard P (2016). River Culture: an eco-social approach to mitigate the biological and cultural diversity crisis in riverscapes. Ecohydrology & Hydrobiology.

