# Supplementary material for: How does a nature-based solution for flood control compare to a technical solution? Case study evidence from Belgium
Source: Ambio. 2021 May 11;50(8):1431–45. doi: 10.1007/s13280-021-01548-4 (PMC8249589; doi:10.1007/s13280-021-01548-4)
Supplement: Supplementary file 1 — Supplementary file1 (PDF 513 KB) [file 13280_2021_1548_MOESM1_ESM.pdf]

Ambio

Electronic Supplementary Material N°1
This supplementary material has not been peer reviewed.

Title: How does a nature-based solution for flood control compare to a technical solution? Case study evidence from Belgium

Authors:

Francis Turkelboom, Rolinde Demeyer, Liesbet Vranken, Piet De Becker, Filip Raymaekers, Lieven De Smet

Online survey with questions to elicit preferences of respondents between a technical and a nature-based solutions in the Dijle Valley

Algemene informatie

Deze enquête peilt naar de voorkeuren van mensen in het gebied 'De Dijlevallei'.
Sinds 1995 is er veel veranderd in het gebied. Aan de hand van dit onderzoek willen wij nagaan wat hiervan het resultaat is.
In deze enquête wordt er gevraagd naar uw voorkeur voor een bepaald scenario, hierbij zit een fictief scenario.
Dit onderzoek betekent niet dat er plannen zijn om dit scenario in het gebied toe te passen, maar de resultaten van dit onderzoek kunnen later eventueel gebruikt worden op andere locaties. In deze enquête wordt er gevraagd naar uw voorkeur voor een bepaald scenario.
Gelieve eerlijk te antwoorden op de vragen.

A. Algemene vragen over 'De Dijlevallei'

De Dijle is een rivier die ontspringt in Houtain-le-Val in Waals-Brabant en komt samen met de Nete tezamen in de buurt van Rumst in de Rupel. Het stuk van de Dijlevallei dat in het onderzoek gebruikt wordt loopt van aan de **taalgrens** tot aan de **Celestijnenlaan** stroomopwaarts van de stad Leuven. In de breedte wordt een strook genomen van ongeveer 1,5 km.

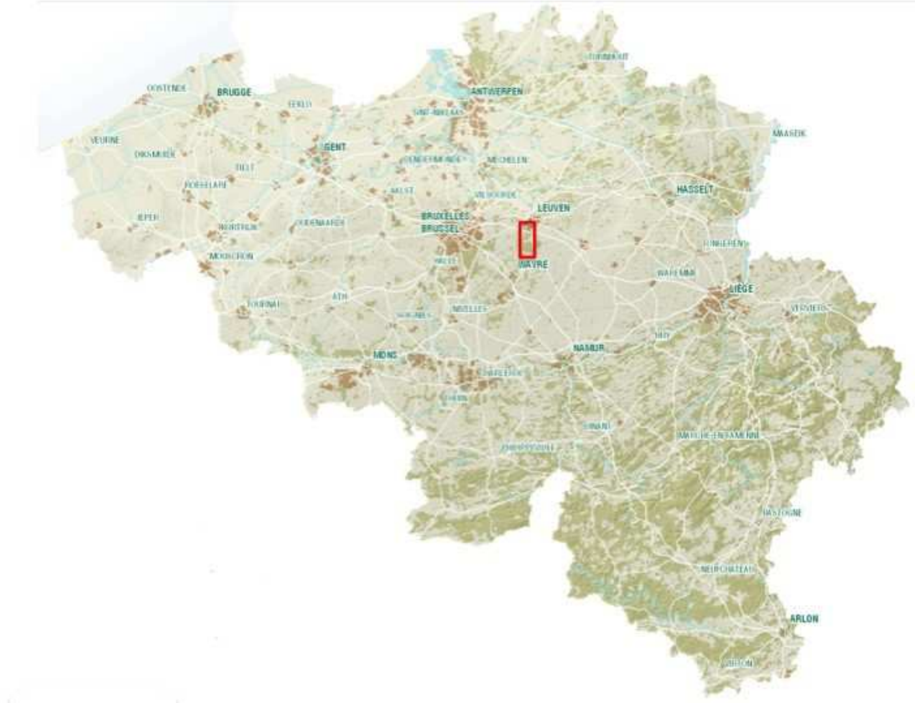

Afbeelding 1: Situering van het studiegebied

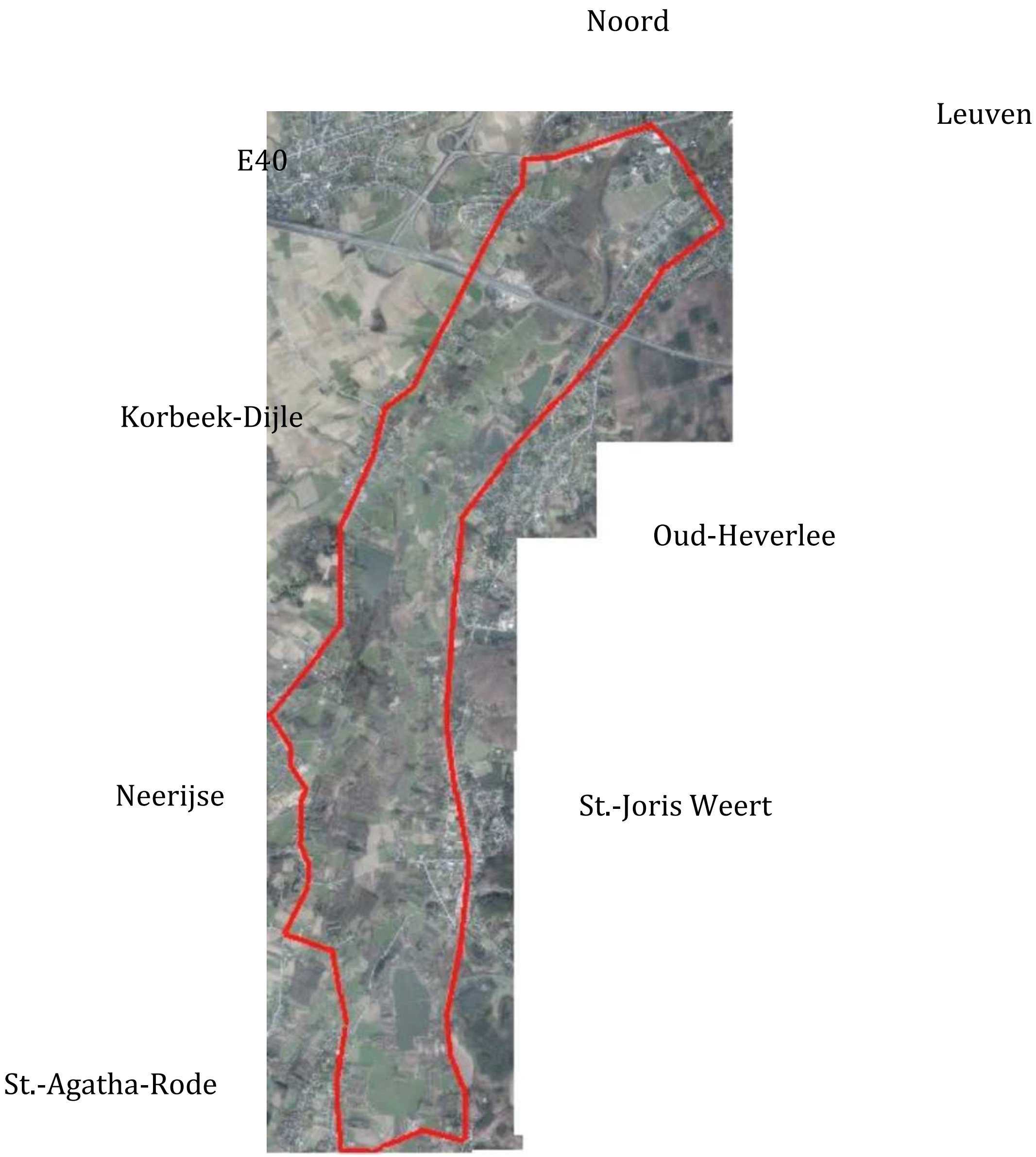

Afbeelding 2: Detailweergave van het studiegebied

1. Hebt u al van het gebied 'De Dijlevallei' gehoord, zoals hoger beschreven, voordat u aan deze enquête deelnam ?

Kies a.u.b. een van de volgende mogelijkheden:

- ☐ Ja
- ☐ Neen

2. Hoe vaak komt u in het gebied 'De Dijlevallei' voor vrijetijdsbesteding?

Kies a.u.b. een van de volgende mogelijkheden:

- ☐ Dagelijks
- ☐ Wekelijks
- ☐ Eens per maand
- ☐ Enkele keren per jaar
- ☐ Ooit eens
- ☐ Nooit
- ☐ Geen mening

3. Welke activiteiten beoefent u als u het gebied 'De Dijlevallei' bezoekt?

Selecteer alles wat voldoet

- ☐ Wandelen
- ☐ Lopen/joggen
- ☐ Fietsen
- ☐ Vissen
- ☐ Bestuderen van de natuur bijv. Vogelkijken
- ☐ Watersport (zwemmen, kajak,…)
- ☐ Andere (specifieer a.u.b.) :

B. Verschillende scenario's voor het gebied 'De Dijlevallei'

Eerst beschrijven we hieronder de verschillende kenmerken die gebruikt worden voor de verschillende scenario's verder in de enquête. In de enquête worden drie verschillende scenario's beschreven. U vindt deze drie scenario's in de bijlage. Aan de hand van een kaart stellen we u telkens twee scenario's voor Stel dat de keuzes op de kaart zich zouden voordoen. De vraag aan u is om aan te geven welk scenario uw voorkeur heeft.

1. **Dijken en oevers**
    a. Natuurlijke oevers van de Dijle, worden regelmatig gemaaid en geruimd.
    b. Betonnen constructies aanwezig
    c. Natuurlijke oevers van de Dijle zonder enig beheer.

2. **Biodiversiteit op het land**
    a. Hoog, er zijn veel soorten aanwezig waaronder ook weinig voorkomende soorten
    b. Laag, er zijn weinig verschillende soorten aanwezig
    c. Matig, er zijn meer soorten aanwezig, maar voornamelijk algemene

3. **Waterkwaliteit en biodiversiteit in het water**
    a. Goed, geen geurhinder of schuim. Verschillende vissoorten en waterplanten aanwezig
    b. Matig, weinig geurhinder of schuim. Enkele vis of waterplanten aanwezig
    c. Slecht, geurhinder en schuim op het water, geen vis of waterplanten aanwezig

4. **Wandel-, fiets- en vogelkijkmogelijkheden**
    a. Meerdere wandel- of fietspaden aanwezig, informatieborden en vogelkijkhutten aanwezig.
    b. Enkele wandel- of fietspaden aanwezig
    c. Nauwelijks wandel- of fietspaden aanwezig

5. **Landschap**
    a. Natuurlandschap, natuurlijke ontwikkeling van de vegetatie, zicht beperkter
    b. Landbouwlandschap, afgebakende beheerde weiden, grote open ruimtes

Er wordt u gevraagd welk bedrag uw gezin wil betalen om het scenario van uw voorkeur te bereiken. Dit bedrag wordt geïnd door middel van een verhoging van de belastingen. Het geld dat via deze belasting wordt geïnd zal in een speciaal fonds voor de ontwikkeling van natuurlijke landschappen verzameld worden en zal enkel voor deze doelen gebruikt worden.
Mogen wij u er nogmaals aan herinneren dat er geen veranderingen in het gebied zullen plaatsvinden. Dit onderzoek dient enkel om te leren voor andere gebieden en zal geen invloed hebben op 'de Dijlevallei'.

1. **a. Verkiest u scenario A of B ?**
   Bijlage 1

   ☐ Scenario A
   ☐ Scenario B

   b. U verkiest scenario A, stel er ligt een plan klaar om naar scenario B te evolueren. Hoeveel geld zou u per maand willen betalen om de realisatie van scenario B tegen te gaan (en dus scenario A te behouden)?

   c. Bent u bereid .. euro/maand te betalen ? Ja/Neen

   d. U verkiest scenario B, stel er ligt een plan klaar om naar scenario B te evolueren. Hoeveel geld zou u per maand willen betalen om scenario B te realiseren?

   e. Bent u bereid .. euro/maand te betalen ? Ja/ Neen

   f. Hoe vaak zou je 'de Dijlevallei' bezoeken voor vrijetijdsbesteding indien scenario B gerealiseerd wordt?
   Kies a.u.b. een van de volgende mogelijkheden:
   - ☐ Dagelijks
   - ☐ Wekelijks
   - ☐ Eens per maand
   - ☐ Enkele keren per jaar
   - ☐ Ooit eens
   - ☐ Nooit
   - ☐ Geen mening

2. **a. Verkiest u scenario A of C ?**
   Bijlage 2

   ☐ Scenario A
   ☐ Scenario C

   b. U verkiest scenario A, stel er ligt een plan klaar om naar scenario C te evolueren. Hoeveel geld zou u per maand willen betalen om de realisatie van scenario B tegen te gaan (en dus scenario A te behouden)?

   c. Bent u bereid .. euro/maand te betalen ? Ja/Neen

   d. U verkiest scenario C, stel er ligt een plan klaar om naar scenario C te evolueren. Hoeveel geld zou u per maand willen betalen om scenario C te realiseren?

   e. Bent u bereid .. euro/maand te betalen ? Ja/Neen

   f. Hoe vaak zou je 'de Dijlevallei' bezoeken voor vrijetijdsbesteding indien scenario C gerealiseerd wordt?
   Kies a.u.b. een van de volgende mogelijkheden:
   - ☐ Dagelijks
   - ☐ Wekelijks
   - ☐ Eens per maand
   - ☐ Enkele keren per jaar
   - ☐ Ooit eens
   - ☐ Nooit
   - ☐ Geen mening

3. **U antwoorde elke keer Neen, kunt u hiervoor de meest passende reden aangeven?**
   ☐ De bedragen zijn te laag
   ☐ Natuur kan niet vervangen worden, voor geen enkel bedrag

C. Socio-demografische karakteristieken

We willen u nu graag een aantal vragen stellen over uzelf. Deze informatie is noodzakelijk voor de verdere analyse van de resultaten. We garanderen u dat al uw antwoorden vertrouwelijk worden behandeld, niet ter beschikking worden gesteld aan derden en niet voor andere doeleinden dan voor dit onderzoek worden gebruikt.

1. Bent u een man of een vrouw?

☐ Man
☐ Vrouw

2. Wat is uw geboortejaar?

....

3. Wat is uw postcode?

....

4. Wat is uw huisadres

....

5. Woonde u vroeger in de buurt van een gebied gelijkaardig aan 'De Dijlevallei'?

Kies a.u.b. een van de volgende mogelijkheden:

☐ Ja
☐ Neen

6. Hoeveel personen telt uw gezin op dit moment (onder hetzelfde dak wonend)?

Vul uw antwoord hier in:

7. Wat is uw hoogst voltooide opleiding?

Kies a.u.b. een van de volgende mogelijkheden:

☐ Middelbaar onderwijs (ASO, TSO, KSO, BSO) of minder
☐ Hogeschool, universitair diploma of hoger

8. Wat is uw huidige werksituatie?

Kies a.u.b. een van de volgende mogelijkheden:

☐ Student

☐ Niet tewerkgesteld (werkzoekende, (brug)pensioen, huisvrouw/man, arbeidsongeschikt, …)

☐ Tewerkgesteld (ambtenaar, bediende, arbeider, zelfstandige,…)

9. In welke categorie valt het maandelijks netto gezinsinkomen (dit is het inkomen van alle inwonende gezinsleden samen, na aftrek van de belastingen)? Deze informatie wordt strikt vertrouwelijk behandeld maar is wel essentieel in de analyse van de resultaten.

Kies a.u.b. een van de volgende mogelijkheden:

☐ Minder dan €500 per maand

☐ €500-750 per maand

☐ €750-1000 per maand

☐ €1000-1250 per maand

☐ €1250-1500 per maand

☐ €1500-2000 per maand

☐ €2000-2500 per maand

☐ €2500-3000 per maand

☐ €3000-3500 per maand

☐ €3500-4000 per maand

☐ €4000-4500 per maand

☐ €4500-5000 per maand

☐ €5000-5500 per maand

☐ €5500-6000 per maand

☐ Meer dan €6000 per maand

☐ Niet van toepassing

10. Bent u of iemand in uw huishouden actief lid of donateur (vorige donatie maximaal een jaar geleden) aan een milieu- en/of natuurorganisatie

☐ Ja

☐ Neen

11. Vond u de vragen van de enquête moeilijk?
Kies a.u.b. een van de volgende mogelijkheden
☐ 1(Niet moeilijk) ☐ 2 ☐ 3 ☐ 4 ☐ 5 (Heel moeilijk)

12.

13. Heeft u specifieke opmerkingen over de vragen?
Vul uw antwoord hier in:

14. Vond u de kaarten met de verschillende scenario's duidelijk?
Kies a.u.b. een van de volgende mogelijkheden
☐ 1(Niet moeilijk) ☐ 2 ☐ 3 ☐ 4 ☐ 5 (Heel moeilijk)

15. Heeft u specifieke opmerkingen over de kaarten met de verschillende scenario's?
Vul uw antwoord hier in:

D. Algemene vragen over het belang van natuurlijke landschappen

Natuurlijke landschappen zijn onder te verdelen in:

- **Natuurgebieden**: dit zijn landschappen die bestaan onafhankelijk van duidelijke menselijke activiteit. Voorbeelden zijn bossen, heidegebieden, rivier/beek-valleien, slikken en schorren, ruigten, moerassen, graslanden, struwelen, kustduinen.
- **Semi-natuurlijke gebieden**: dit zijn landschappen die bestaan uit natuurlijke elementen, maar waar een duidelijke invloed van menselijke activiteit te zien is. Voorbeelden zijn akkers, weiden, parken en tuinen, bomenrijen, boomgaarden, hagen.

1. In welke mate vindt u onderstaande thema's belangrijk?
Kies het best passende antwoord voor elk onderdeel

	Erg belangrijk	Belangrijk	Neutraal	Niet belangrijk	Helemaal niet belangrijk	Geen mening
A. Verbeteren van waterkwaliteit	○	○	○	○	○	○
B. Tegengaan van klimaatsverandering	○	○	○	○	○	○
C. Het behoud van bedreigde dier- en plantensoorten	○	○	○	○	○	○
D. Verbeteren van de luchtkwaliteit	○	○	○	○	○	○
E. Verbeteren van het afvalbeleid	○	○	○	○	○	○
F. Verminderen geluidshinder	○	○	○	○	○	○
G. Verminderen geurhinder	○	○	○	○	○	○
H. Vermeerderen van groene gebieden	○	○	○	○	○	○

2. **a. Wanneer je voor vrijetijdsbesteding naar een natuur- of semi-natuurgebied gaat, hoe vaak gaat u naar een gebied binnen een straal van 5 km? _____________ per jaar**

   b. Welk vervoersmiddel gebruikt u dan het vaakst?
   Kies a.u.b. een van de volgende mogelijkheden
   - ☐ Te voet
   - ☐ Per fiets
   - ☐ Auto/moto
   - ☐ Openbaar vervoer
   - ☐ Ander (specifieer a.u.b.) :

   c. Welke activiteit voert u er dan hoofdzakelijk uit?
   Selecteer alles wat voldoet
   - ☐ Wandelen
   - ☐ Lopen/joggen
   - ☐ Fietsen
   - ☐ Vissen
   - ☐ Natuur bestuderen (vb. Vogelkijken)
   - ☐ Watersport (zwemmen, kajak,..)
   - ☐ Andere (specifieer a.u.b.) :

3. **a. Wanneer je voor vrijetijdsbesteding naar een natuur of semi-natuurgebied gaat, hoe vaak gaat u naar een gebied binnen een straal van 20 km? _____________ per jaar**

   b. Welk vervoersmiddel gebruikt u dan het vaakst?
   Kies a.u.b. een van de volgende mogelijkheden
   - ☐ Te voet
   - ☐ Per fiets
   - ☐ Auto/moto
   - ☐ Openbaar vervoer
   - ☐ Andere (specifieer a.u.b.) :

c. Welke activiteit voert u er dan hoofdzakelijk uit?
Selecteer alles wat voldoet

- ☐ Wandelen
- ☐ Lopen/joggen
- ☐ Fietsen
- ☐ Vissen
- ☐ Natuur bestuderen (vb. Vogelkijken)
- ☐ Watersport (zwemmen, kajak,..)
- ☐ Andere (specifieer a.u.b.) :

4. a. Wanneer je voor vrijetijdsbesteding naar een natuur- of semi-natuurgebied gaat, hoe vaak gaat u naar een gebied binnen een straal van 40 km? ______________ per jaar

b. Welk vervoersmiddel gebruikt u dan het vaakst?
Kies a.u.b. een van de volgende mogelijkheden

- ☐ Te voet
- ☐ Per fiets
- ☐ Auto/moto
- ☐ Openbaar vervoer
- ☐ Andere (specifieer a.u.b.) :

c. Welke activiteit voert u er dan hoofdzakelijk uit?
Selecteer alles wat voldoet

- ☐ Wandelen
- ☐ Lopen/joggen
- ☐ Fietsen
- ☐ Vissen
- ☐ Natuur bestuderen (vb. Vogelkijken)
- ☐ Watersport (zwemmen, kajak,..)
- ☐ Andere (specifieer a.u.b.) :

4. In welke mate gaat u akkoord met de volgende stellingen

	Helemaal niet akkoord	Niet akkoord	Eerder niet akkoord	Eerder akkoord	Akkoord	Helemaal akkoord	Geen mening
A. Er zijn voldoende natuurlijke landschappen in mijn onmiddellijke omgeving<br>• **binnen 1 km**	○	○	○	○	○	○	○
• **binnen 5 km**	○	○	○	○	○	○	○
B. Er is voldoende natuur in mijn omgeving<br>• **binnen 1 km**	○	○	○	○	○	○	○
• **binnen 5 km**	○	○	○	○	○	○	○
C. Er is voldoende groene open ruimte (akker-, weiland, park,..) in mijn onmiddellijke omgeving<br>• **binnen 1 km**	○	○	○	○	○	○	○
• **binnen 5 km**	○	○	○	○	○	○	○
D. Bij de keuze van mijn woonplaats speelde de aanwezigheid van natuurlijke landschappen een belangrijke rol	○	○	○	○	○	○	○
E. Alles bij elkaar genomen ben ik tegenwoordig tevreden met mijn woonomgeving	○	○	○	○	○	○	○

E. Dynamische map

Bedankt voor uw deelname!
